# Harnessing evolution: leveraging bacterial isoprenoid pathway diversity toward improved bioengineering strategies

**DOI:** 10.1128/jb.00441-25

**Published:** 2026-02-17

**Authors:** Christine M. Qabar, Bailey A. Marshall, Robert Landick, Jeffery S. Cox

**Affiliations:** 1 Department of Molecular and Cell Biology, University of California Berkeley 196203, Berkeley, California, USA; 2 Department of Biochemistry, University of Wisconsin-Madison 200878, Madison, Wisconsin, USA; 3 DOE Great Lakes Bioenergy Research Center, University of Wisconsin-Madison 5228, Madison, Wisconsin, USA; 4 Department of Bacteriology, University of Wisconsin-Madison 205263, Madison, Wisconsin, USA; National Institutes of Health, Bethesda, Maryland, USA

**Keywords:** isoprenoid metabolism, isoprenoids, terpenoids, metabolism, isoprenoid biosynthesis, microbial engineering, synthetic biology, heterogeneity, MEV, MEP

## Abstract

Isoprenoids play vital roles in all domains of life, from beta-carotene in bacteria to heme in humans. Two distinct metabolic pathways have evolved to synthesize the critical precursor of all mature isoprenoids: the mevalonate (MEV) and the methylerythritol phosphate (MEP) pathways. Here, we quantify the extensive inter- and intra-genus heterogeneity in the usage of these two pathways with particular emphasis on rare bacteria that encode both, or neither, pathways. Furthermore, MEP intermediates themselves have non-isoprenogenic roles that may underlie evolutionary pressures driving pathway diversification. Understanding isoprenoid biosynthesis in bacteria offers new avenues toward more sustainable engineering of economically relevant molecules in microbes.

## ISOPRENOIDS ARE UBIQUITOUS AND ESSENTIAL ACROSS THE DOMAINS OF LIFE

### Isoprenoids are a massive and diverse class of biomolecules

Isoprenoids, also referred to as terpenoids, are a broad and diverse class of molecules, including ~95,000 different compounds that have been identified to date (1, 2). Defined by repeating five-carbon isoprene units, these compounds play crucial roles in various processes in all domains of life, including ATP synthesis, protein modification, hormone signaling, maintenance of membrane structure and fluidity, pigment production, and stress response (3, 4). Key isoprenoids produced in animals include heme, cholesterol, steroids, and vitamin D, as well as post-translational prenyl or farnesyl modifications (5, 6). Furthermore, archaeal membranes contain essential isoprenoid components (7), and fungal isoprenoids are pharmacologically valuable as antimicrobials and antitumors (8). In addition to mediating basic cellular functions, plant isoprenoids have been used in indigenous cultures for thousands of years and are now being isolated for use in medicine, agriculture, and cosmetics (9). Although isoprenoids are integral in all domains of life, we will focus on bacteria as this kingdom contains the most diverse range in isoprenoid biosynthesis strategies. Importantly, this existing natural diversity can be harnessed in ongoing efforts to bioengineer isoprenoids at scale in bacteria.

### Isoprenoids participate directly and indirectly in myriad bacterial processes

In bacteria, isoprenoids directly support both vital cellular processes, such as cell wall synthesis and electron transport, as well as secondary bacterial behaviors, including intermicrobial signaling and host-microbe interactions (3, 4, 10). For example, the critical electron carriers, ubiquinone and menaquinone, have isoprenoid modifications, and isoprenoid lipids, called hopanoids, have been shown to support resistance to pH, bile salt, and antibiotic stress in some bacteria (4). For further reading on the direct effects of isoprenoid biosynthesis in bacterial pathogens and more broadly in microbial ecology, we recommend the reviews of Heuston et al. (11) and Avalos et al. (4).

**Fig 1:**
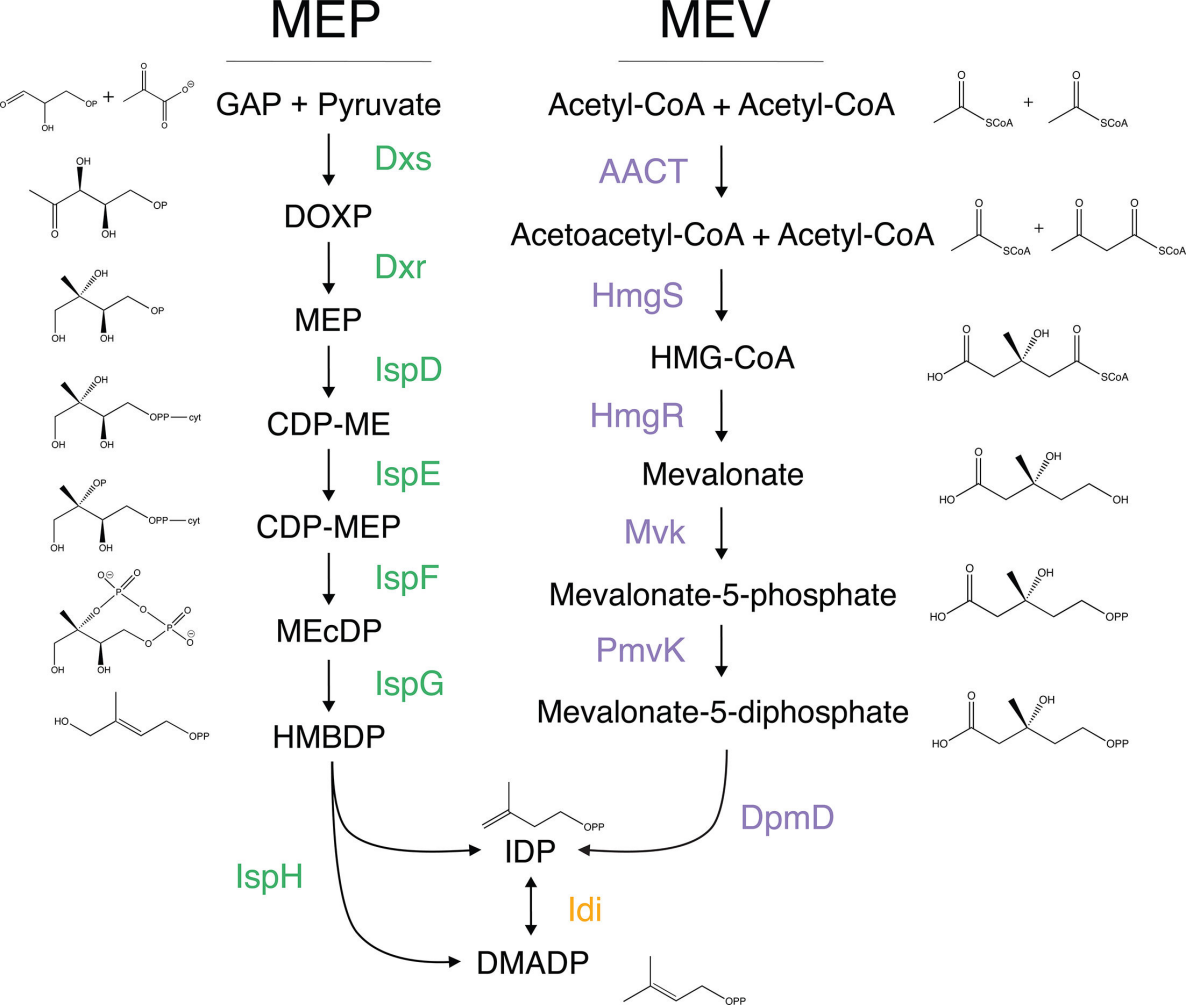
Two pathways of isoprenoid biosynthesis in bacteria. Shown are intermediates and enzymes of the MEV (purple) and MEP (green) pathways of isoprenoid biosynthesis. Orange indicates that the enzyme is found in both pathways. GAP, glyceraldehyde 3-phosphate; Dxs, DOXP synthase; DOXP, 1-deoxyxylulose 5-phosphate; Dxr, DOXP reductoisomerase; MEP, 2-C-methyl-erythritol 4-phosphate; IspD, 2-C-methyl-D-erythritol 4-phosphate cytidylyltransferase; CDP-ME, 4-(cytidine 5′-diphospho)−2-C-methyl-erythritol; IspE, 4-diphosphocytidyl-2-C-methyl-D-erythritol kinase; CDP-MEP, 2-phospho-4-(cytidine 5′-diphospho)−2-C-methyl-erythritol; IspF, 2-C-methyl-D-erythritol 2,4-cyclodiphosphate synthase; MEcDP, 2-C-methyl-erythritol-2,4-cyclodiphosphate; IspG, 4-hydroxy-3-methylbut-2-en-1-yl diphosphate synthase; HMBDP, 4-hydroxy-3-methyl 2-butenyl diphosphate; IspH, 4-hydroxy-3-methylbut-2-enyl diphosphate reductase; IDP, isopentenyl diphosphate; Idi, isopentenyl diphosphate isomerase; DMADP, dimethylallyl diphosphate; AACT, Acetoacetyl-CoA thiolase; HmgS, HMG-CoA synthase; HMG-CoA, hydroxymethylglutaryl-CoA; HmgR, HMG-CoA reductase; Mvk, mevalonate kinase; PmvK, phosphomevalonate kinase; and DpmD, diphosphomevalonate decarboxylase.

Surprisingly, recent work has shed light on the important indirect effects of biosynthetic intermediates on cells, which mediate non-isoprenogenic interactions that are independent of isoprenoid biosynthesis (12, 13). Understanding non-isoprenogenic roles of isoprenoid intermediates expands our understanding of the global cellular effects of isoprenoid biosynthesis, as well as the forces guiding pathway evolution. For example, MEcDP and HMBDP are small molecule intermediates of isoprenoid biosynthesis (Fig. 1) that have been implicated in non-isoprenogenic processes such as oxidative stress sensing and host immune sensing, further discussed next.

## THE MEP AND MEV PATHWAYS FOR ISOPRENOID BIOSYNTHESIS

### Bacteria can use two pathways to synthesize isoprenoids

**Fig 2:**
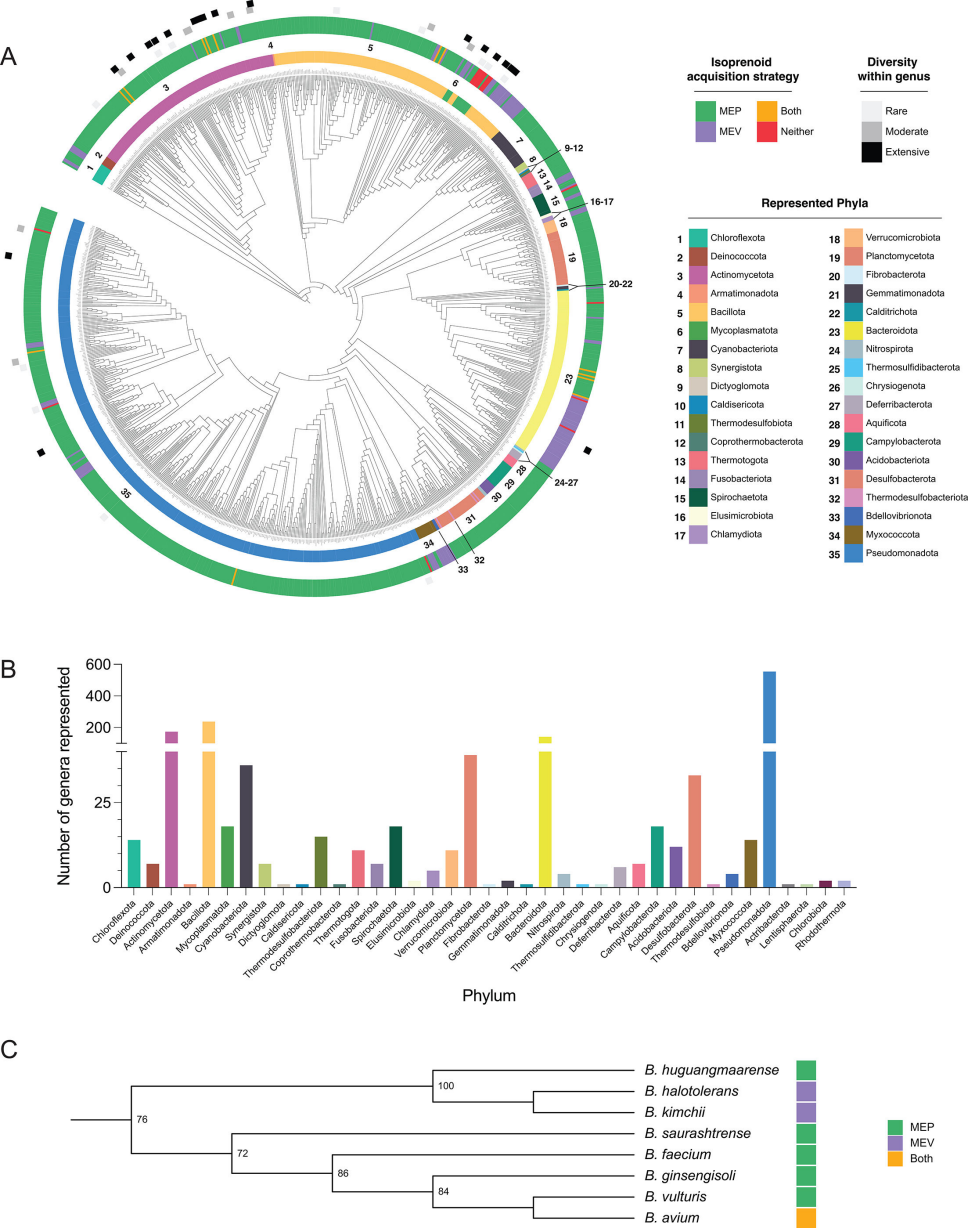
Extensive diversity in isoprenoid acquisition strategies among bacteria. (**A**) The identity of isoprenoid biosynthesis pathways encoded by each genus is shown in the outer ring: MEP (green), MEV (purple), neither (red), or both (orange). Diversity within a genus is indicated by squares in light gray (rare exceptions, <10% of total species diverge from the primary genus call), dark gray (moderate diversity, 10%–25% of the total species diverge from the genus call), or black (extensive diversity, >25% of total species diverge from the genus call). Phyla are indicated by the numbered inner ring. The data shown represent 1,033 unique genera and 36 unique phyla. The Genome Taxonomy Database (GTDB) Bac120 tree (16) was visualized using iTol v7 (17) and pruned to remove unnamed isolates and display one node per genus. The NCBI Taxonomy and GTDB databases were used to assign phyla, and previously published homology identification (18) was used to make isoprenoid strategy and diversity calls. iTol Tree ID 108654132457551738254768. (**B**) Genus-level representation in pathway analysis. Table S1 is an expanded, species-level analysis of isoprenoid pathway usage. Analysis includes 4,249 species, representing 1,412 genera and 39 phyla. (**C**) Brachybacterium, within the phylum Actinomycetota, contains species with different isoprenoid biosynthesis strategies. As in panel **A**, the pathways encoded by each species are noted in green (MEP), purple (MEV), or orange (both). 16s rRNA sequences were acquired from the NCBI Nucleotide database (19). Geneious Prime (v2024.0.7) was used to perform a MUSCLE alignment (20) and build a PhyML tree (21) with 50 bootstraps. Annotation and tree visualization were performed on iTol (17). iTol Tree ID 10865413219911738351554.

Isopentenyl diphosphate (IDP) and its isomer dimethylallyl diphosphate (DMADP) are the five-carbon precursor units used to build all isoprenoids. Two distinct pathways exist to generate these precursors: the mevalonate (MEV, sometimes referred to as the MVA) pathway and the methylerythritol phosphate (MEP, also known as the non-mevalonate) pathway (Fig. 1). Although the MEP and MEV pathways both generate the same end products, they are not simply interchangeable; they proceed via different enzymatic steps and thus have unique cofactor and energetic requirements, as well as unique non-isoprenogenic effects from metabolic intermediate interactions (Table 1). For example, the MEP pathway is more carbon-efficient as it requires fewer glyceraldehyde-3-phosphate molecules per IDP/DMADP produced, while the MEV pathway is more energy-efficient because it generates a net yield of reducing equivalents (14, 15). The distribution of these pathways has largely been regarded as mutually exclusive, barring a few exceptions; however, in this review, we bring into focus the extensive diversity of pathway utilization even within groups of closely related bacteria (Fig. 2). Understanding the evolutionary forces that influence pathway usage may inform engineering efforts that aspire to maximize isoprenoid production for sustainable human use, which is explored in “Synthetic biology approaches to engineer isoprenoid biosynthesis,” below.

**TABLE 1:** Enzymatic steps of the MEP and MEV pathways^*a*^^,^^*b*^

Pathway	Enzyme	Reaction	Other features
MEP pathway	DOXP synthase (**Dxs**; EC 2.2.1.7)	Condensation of pyruvate + D-glyceraldehyde 3-phosphate → 1-deoxy-D-xylulose 5-phosphate (**DOXP**)	Cofactors: Mg^2+^, thiamin diphosphate (TDP). Rate-limiting step of MEP. DOXP can be shunted to thiamin, pyridoxal biosynthesis (22). Feedback inhibited by IDP, DMADP (23). Enhanced in the presence of GAP (24).
	DOXP reductoisomerase (**Dxr**; EC 1.1.1.267)	Reversible rearrangement from DOXP → 2-C-methylerythritol 4-phosphate (**MEP**)	Cofactors: divalent cation (Co^2+^, Mn^2+^, Mg^2+^), NADPH. Types I and II Dxr have scattered taxonomic distribution (25) and are both targeted by fosmidomycin (26). First committed step of the MEP pathway (27).
	MEP cytidylyltransferase (**IspD**; EC 2.7.7.60)	MEP → 4-diphosphocytidyl-2-C-methylerythritol (**CDP-ME**)	Cofactors: CTP, Mg^2+^. IspDF fusion proteins are common, despite catalyzing nonconsecutive steps (28).
	CDP-ME kinase (**IspE**; EC 2.7.1.148)	CDP-ME → 4-diphosphocytidyl- 2-C-methyl-D-erythritol 2-phosphate (**CDP-MEP**)	Cofactor: ATP.
	MEcDP synthase (**IspF**; EC 4.6.1.12)	CDP-MEP → 2-C-methyl-D-erythritol 2,4-cyclodiphosphate (**MEcDP**)	Cofactors: Zn^2+^ AND Mg^2+^. Farnesyl diphosphate (FDP) inhibits *E. coli* IspF-MEP complex, but stabilizes free IspF (29). MEcDP accumulates under oxidative stress (12).
	HMBDP synthase (**IspG, GcpE**; EC 1.17.7.3)	MEcDP→ (E)−4-Hydroxy-3-methyl-but-2-enyl diphosphate (**HMBDP**)	Cofactors: 4Fe-4S, NADPH, reducing equivalent (flavodoxin). HMBDP activates Vγ9Vδ2 T cells (30) and accumulates under nitrosative stress in *M. smegmatis* (31). Susceptible to oxidative damage (32).
	HMBDP reductase (**IspH, LytB**; EC 1.17.7.4)	HMBDP → isopentenyl diphosphate (**IDP**) and dimethylallyl diphosphate (**DMADP**)	Cofactors: FAD, divalent cations (Co^2+^), 4Fe-4S, NADPH, reducing equivalent (flavodoxin or ferredoxin). Makes 5:1 IDP:DMADP (33), although this ratio is variable based on the enzyme and its expression (34). Reported substrate promiscuity (35). Susceptible to oxidative damage (32).
Both	Isopentenyl diphosphate isomerase (**Idi**; EC 5.3.3.2)	Isomerization of IDP ⇌ DMADP	Cofactors: Zn^2+^, Mg^2+^ (**IDI-1**) or Mg^2+^, FMN, NADPH (**IDI-2**). Types I (bacteria, eukaryotes) and II (bacteria, archaea) Idi are structurally unrelated (36). No correlation between Idi type and MEV vs MEP pathway usage (10).
MEV pathway	Acetoacetyl-CoA thiolase (**AACT**; EC 2.3.1.9)	Condensation of 2 acetyl-CoA → acetoacetyl-CoA	*E. faecalis mvaE* encodes both AACT and HmgR (37).
	HMG-CoA synthase (**HmgS**; EC 2.3.3.10)	Condensation of acetoacetyl-CoA + acetyl-CoA → 3-hydroxy-3-methylglutaryl-CoA (**HMG-CoA**)	Regulated by sterols (5).
	HMG-CoA reductase (**HmgR**; EC 1.1.1.34)	HMG-CoA → mevalonate	Rate-limiting step of MEV. Regulated by sterols (38). Class I (archaea, eukaryotes, and some bacteria) is inhibited by statin drugs and uses NADPH, while class II (bacteria) is less sensitive to statins and varies in the use of NADPH or NADH as a cofactor (39–42).
	Mevalonate kinase (**Mvk**; EC 2.7.1.36)	Mevalonate → mevalonate-5-phosphate	Cofactors: Mg^2+^, ATP. Feedback is inhibited by downstream isoprenoids (class I) (43, 44) and mevalonate-5-diphosphate (class II) (45).
	Phosphomevalonate kinase (**PmvK**; EC 2.7.4.2)	Mevalonate-5-phosphate → mevalonate-5-diphosphate	Cofactors: Mg^2+^, ATP.
	Diphosphomevalonate decarboxylase (**DpmD**; EC 4.1.1.33)	Mevalonate-5-diphosphate → IDP	Cofactors: Mg^2+^, ATP. Generates only IDP, unlike the terminal enzyme of the MEP pathway.

^*a*^ Bold text indicates enzymes referenced in the main text.

^*b*^ Gray shading indicates enzymes shared between both pathways.

### The MEP pathway is the predominant bacterial isoprenoid biosynthesis pathway

The MEP pathway of isoprenoid biosynthesis is found in most but not all bacteria (11, 18, 46), as well as in plant plastids and apicomplexan parasites like *Toxoplasma gondii* and *Plasmodium* spp (27). Interestingly, plants have both pathways spatially separated in organelles, with cytosolic IDP production occurring via the MEV pathway and plastidal synthesis occurring via the MEP pathway, a segregation likely originating from an early bacterial endosymbiosis event (38, 47, 48). Furthermore, apicomplexans only encode the MEP pathway in the genome of their apicoplast, an organelle that is cyanobacterial in origin (49). The mechanistic studies that identified MEP enzymes have been reviewed in detail (32, 50–52), and the regulation of this pathway has been most often reviewed in plants (53). The persistence of both isoprenoid biosynthesis pathways across the kingdoms of life and the partitioning of the MEP pathway within organelles indicate selective pressure for each pathway in certain conditions.

Isoprenoid biosynthesis via the MEP pathway generates both IDP and DMADP via seven enzymatic steps and uses 2–3 NADPH depending on isozyme usage, 1 CTP, 1 ATP, and a reducing partner, which may be ferredoxin or flavodoxin (Fig. 1). This pathway is heavily regulated through a network of feedback mechanisms but has a higher theoretical yield than the MEV pathway (54). The tightly regulated MEP pathway branches off from glycolysis via the condensation of pyruvate and D-glyceraldehyde 3-phosphate to produce DOXP by the enzyme Dxs. This is the rate-limiting step of the pathway and is feedback inhibited by IDP/DMADP binding of Dxs, thus serving as the main control point of MEP flux (23, 55). Next, Dxr rearranges DOXP to form MEP, the first committed intermediate for which the pathway is named. The subsequent three steps mediated by IspD, IspE, and IspF activate and cyclize MEP to form MEcDP, an important intermediate which is discussed later in this section. The FeS cluster-containing protein IspG (also referred to as GcpE) then catalyzes the conversion of MEcDP to HMBDP, an intermediate that potently stimulates an immune response in the context of infection (30). IspH (also referred to as LytB) then converts HMBDP to IDP and DMADP at a ratio favoring IDP, although this ratio is variable based on the enzyme of study (34). Understanding this diversity in enzyme function is of interest from a bioengineering perspective; however, the mechanistic underpinnings of this phenomenon are not well understood. Interestingly, while the first step catalyzed by Dxs is rate-limiting, both IspG and IspH are considered important pathway bottlenecks due to their redox-sensitive FeS clusters (56, 57). Finally, IDP and DMADP can be interconverted via the enzyme Idi (Fig. 1).

### The MEV pathway is used by some bacteria and is predominant in eukaryotes and archaea

The mevalonate pathway of isoprenoid biosynthesis has been reviewed extensively in eukaryotes (5, 39, 58) and archaea (59, 60) and is of particular interest in human medicine, as statin drugs that target this pathway are routinely used to lower cholesterol and synergize with cancer therapy (61). In the bacterial kingdom, the MEV pathway is less ubiquitous than the MEP pathway but is still used by some bacteria, including *Borrelia burgdorferi*, *Staphylococcus aureus*, and *Streptococcus pneumoniae* (39).

The MEV pathway consists of six enzymatic steps and uses two NADPHs and three ATPs (Fig. 1). First, two acetyl-CoA molecules are condensed to produce acetoacetyl-CoA by the enzyme acetoacetyl-CoA thiolase. HmgS then condenses acetoacetyl-CoA with another molecule of acetyl-CoA to generate Hmg-CoA, which is reduced by the rate-limiting enzyme HmgR to produce mevalonate, for which this pathway is named (62). Two sequential phosphorylation reactions by the kinases Mvk and Pmvk generate mevalonate-5-diphosphate, which is then converted to IDP by the enzyme Dpmd. In contrast to the MEP pathway, the MEV pathway generates only IDP and requires Idi to isomerize IDP to DMADP.

There is a eukaryotic/bacterial-type MEV pathway and an archaeal-type MEV pathway, which differ in the intermediate steps taken, but ultimately both lead to the generation of IDP and DMADP (40, 63). Furthermore, while the bacterial MEV pathway is most similar to that of eukaryotes, some members of the phylum Chloroflexota encode an archaeal-type MEV pathway (64, 65), supporting a model in which multiple mechanisms of MEV gene acquisition occurred in bacteria. Clearly, there exists considerable heterogeneity even among organisms that use the MEV pathway, presenting a fascinating question: how did this pathway evolve?

### Origins and evolution of isoprenoid biosynthesis pathways

The evolutionary origin of isoprenoid biosynthetic pathways remains controversial. It is widely accepted that the MEV pathway is ancestral in all domains of life and that the MEP pathway subsequently evolved in bacteria. However, there is disagreement on whether MEV emerged in the last universal common ancestor or, as new phylogeny work suggests, from the newly discovered *Asgardarchaeota* phylum, which is thought to be ancestral to eukaryotes (63). Even in bacteria, the origin of the MEV pathway remains unresolved. Some suggest that certain bacterial lineages retained MEV genes from the last universal common ancestor (58), while others argue that these genes were horizontally acquired from eukaryotes (3).

While the precise origin of the bacterial MEV pathway remains elusive, the evolutionary course of isoprenoid biosynthesis in bacteria likely unfolded through multiple routes from potentially multiple ancestors. What is clear is that there is considerable genetic, metabolic, and enzymatic diversity to all arrive at the same molecule: IDP. The distinct regulation and energetic requirements of these two pathways were likely critical drivers of this diversification. However, it is possible that the variability in the non-isoprenogenic roles of each pathway, especially of their intermediates, has played a role in the evolutionary pressure to develop multiple routes for isoprenoid biosynthesis.

### Gene- and protein-level diversity

Our understanding of the evolutionary course of isoprenoid biosynthesis pathways is complicated by the phylogenies of individual enzymes. For example, HmgR, primarily found in archaea, eukaryotes, and some bacteria (type I), is distinct from those present mostly in bacteria, and a few archaea (type II), and the two are differentially susceptible to statin drugs (10, 58).

Idi presents another node of variability in both the MEP and MEV pathways. It is the only shared enzyme between both the MEV and MEP pathways and is found in two forms that are structurally unrelated and have different cofactor requirements (36). Bacteria possess either the eukaryotic type I Idi or the archaeal type II Idi, with no correlation between pathway usage and Idi type (10). Some bacteria, including *Mycobacterium marinum*, encode both type I and II Idi enzymes (36).

Gene-level diversity also exists in the MEP pathway, further demonstrating considerable flexibility within this essential biosynthetic process. The first committed step of the MEP path is carried out by the enzyme Dxr, which exists in two forms: Dxr-I and Dxr-II, which only share sequence-level homology at the NADPH-binding domain (10). Type II Dxr is sporadically distributed across the bacterial kingdom in a manner that cannot be easily explained by HGT and instead is thought to have occurred through gene loss from a complex ancestor (66). No defining pattern of Dxr distribution among bacteria has emerged other than overrepresentation among certain pathogens (25). The existence of gene-level variance in both MEP and MEV genes suggests that these pathways are quite flexible, allowing for specialization and survival in a broad range of niches.

There are instances of organisms that use enzymatic steps that are slightly different from the canonical pathways to generate IDP (40, 67). These alternative “routes” still possess the namesake metabolic intermediate of each pathway, methylerythritol phosphate (MEP) or mevalonate (MEV). For example, three routes through the MEV pathway have been identified in archaea, including some with feedback-resistant enzymes, suggesting divergent mechanisms of regulation across the domains of life (68–70). Regarding the MEP pathway, several mutations outside of the MEP pathway have been identified that allow for the suppression of Dxr- and Dxs-knockout lethality, suggesting that there are functional, non-canonical routes through this pathway (71–73). Taken together, this flexibility indicates that although isoprenoid biosynthesis is essential, these pathways are highly adapted to the metabolic demands of a given niche.

Although the ancestral emergence of the MEV pathway is incompletely resolved, the current diversity of isoprenoid biosynthesis strategies reflects the breadth of metabolic requirements and capacities in bacteria. This is especially apparent in the distribution of these strategies across the bacterial kingdom (Fig. 2A). Furthermore, the heterogeneity of pathway types, enzyme classes, and evolutionary routes clearly demonstrates the genetic and mechanistic plasticity of isoprenoid biosynthesis across the domains of life, which provides a rich source of inspiration for metabolic engineers to effectively manipulate isoprenoid biosynthesis.

### MEP pathway intermediates have non-isoprenogenic roles

#### MEcDP may serve as an oxidative stress signaling molecule

There is growing evidence that the MEP pathway intermediate MEcDP (Fig. 1) acts as an oxidative stress sensor because it accumulates under oxidative stress, acts as a stress signaling molecule in plants, and is remarkably stable due to its cyclized-diphosphate structure, which is characteristic of signaling molecules (12, 31, 74, 75). Interestingly, the pathogen *L. monocytogenes* and the nonpathogenic *L. innocua* encode genes for both the MEP and MEV pathways, although IspG and IspH are completely absent in *L. innocua* and IspG is defective in *L. monocytogenes* (76, 77). Importantly, the partial MEP pathway encoded by these species is functional and produces MEcDP (78), suggesting that this pathway might primarily serve to produce this molecule to signal oxidative stress. Furthermore, the oxygen-sensitive MEcDP synthase IspF was determined to be important to oxidative stress resistance in *L. monocytogenes*, and IspF, IspG, and IspH all have roles in murine infection despite this pathogen primarily using the MEV pathway for isoprenoid biosynthesis (76, 79, 80). It appears that the MEV pathway in *Listeria* supplies the IDP needed for isoprenoid production, while the MEP pathway serves as a metabolic stress response system, supporting a model in which isoprenoid biosynthesis pathway diversity enables environmental flexibility. In this model, the MEP pathway role is non-isoprenogenic in *Listeria* and is instead used to signal oxidative stress and provide an adaptive advantage.

#### HMBDP is recognized by immune cells

Another critical role mediated by an MEP intermediate is found in HMBDP (also referred to as HMBPP), which is the most potent and specific activator of Vγ9Vδ2 T cells, a specialized subset of γδ T cells in primates (30). While these non-canonical T cells can also respond to host-derived IDP, HMBDP is a 10,000-fold more potent activator (30), strongly suggesting that this sensing pathway represents a self-nonself detection mechanism. Importantly, Vγ9Vδ2 T cells expand significantly in response to infection by HMBDP-producing pathogens, including *Mycobacterium tuberculosis, L. monocytogenes,* and *Plasmodium spp*. (13). Once activated, Vγ9Vδ2 T cells mount a robust immune response characterized by the secretion of antimicrobial factors, polarization of αβ T cells, and establishment of immune memory (81). Thus, the Vγ9Vδ2 T cell response appears to be a robust and dedicated mechanism by which the host can detect bacteria via HMBDP binding and is a promising vaccine strategy against *M. tuberculosis*.

The Vγ9Vδ2 T cell response is protective in the context of infection but may also be involved in symbiotic bacterial-host homeostasis. It has been suggested in the literature that some pathogens evolved to use only the MEV pathway as an immune evasion strategy; however, the distribution of either pathway in successful pathogens and commensals alike suggests that host immunity was not the primary evolutionary pressure responsible for pathway diversification in bacteria (11). While MEV-utilizing bacteria do not stimulate a Vγ9Vδ2 T cell response, there are successful pathogens that necessarily use the MEP pathway, including *M. tuberculosis*. It is more likely that other factors, including virulence programs and intrinsic resistance, play a larger role in pathogenesis than the utilization of either pathway of isoprenoid biosynthesis. Rather, HMBDP sensing by Vγ9Vδ2 T cells may have evolved as a recent adaptation by primates to communicate with their bacterial symbionts, representing a unique mechanism of host detection of MEP-utilizing microbes.

## ISOPRENOID PATHWAY DIVERSITY IN BACTERIA

### Bacteria with an incomplete endogenous isoprenoid biosynthesis pathway

Although it is essential that cells have a strategy to obtain IDP, some bacteria have evolved to have an incomplete isoprenoid biosynthesis pathway or to lack one altogether. These auxotrophs must then acquire precursors or mature isoprenoids from prototrophs, resulting in an obligate symbiosis. For example, members of the class Mollicute are often symbionts of eukaryotic organisms and have reduced metabolic capabilities due to genome reduction. As Mollicutes streamline their genome to the bare essential genes for survival with their hosts, they often forego a primary isoprenoid biosynthesis strategy, as in the case of the human pathogen *Mycoplasma genitalium,* which possesses neither the MEP nor the MEV pathway (82, 83). Instead, *M. genitalium’s* isoprenoid acquisition strategy is to steal precursors or mature isoprenoids from its host.

Even as some bacteria have lost independent isoprenoid biosynthesis capabilities, their close relatives may retain a partial pathway, likely for non-isoprenogenic reasons. A systematic analysis of isoprenoid biosynthesis enzymes in the available repository of bacterial genomes revealed 72 other Mollicute species from the genera *Mesomycoplasma*, *Mesoplasma*, *Metamycoplasma*, *Mycoplasma*, *Mycoplasmoides*, and *Mycoplasmopsis* that lack homologs for isoprenoid biosynthesis genes (18). Interestingly, two notable exceptions emerged: *Mycoplasma tullyi* and *Mycoplasmoides gallisepticum*, in which all but the final MEP enzymes were identified despite having very small genomes at 0.867 and 0.964 Mb, respectively, and whose close relatives have totally dispensed with these genes (18, 84, 85). The presence of a near-complete MEP pathway in an ultra-reduced genomic landscape raises fascinating questions about the evolutionary trajectory of these bacteria and further suggests that the non-isoprenogenic functions of the MEP pathway may drive such diversification.

Endogenous isoprenoid biosynthesis pathways with varying degrees of completeness have been identified in yet more Mollicutes, underscoring the heterogeneity within this class. For example, most host-associated *Spiroplasma* possess complete MEP pathways, while one species lacks the critical enzyme Dxs and two species lack any MEP genes at all (18). Furthermore, most obligate intracellular *Rickettsia* and *Orientia* rely on host-generated isoprenoids (86, 87), although IspD was detected in several *Rickettsia* species (18). Importantly, an intracellular or otherwise host-associated lifestyle does not indicate an incomplete isoprenoid biosynthesis pathway, as demonstrated by *Listeria monocytogenes*, *Shigella flexneri*, and *Francisella tularensis*, which can reside in host cytosols but encode complete isoprenoid biosynthesis pathways. Additionally, genome size is not a definitive predictor of isoprenoid biosynthetic capability; while *Francisella tularensis* (1.9 Mb genome) possesses a complete isoprenoid biosynthesis pathway, *Orientia tsutsugamushi* (2.1 Mb genome) does not and acquires isoprenoids from its host.

While many obligate symbionts encode incomplete pathways or lack them altogether, the detection of isoprenoid biosynthesis genes that do not amount to a complete pathway in ultra-reduced genomes is indicative of extensive heterogeneity in isoprenoid acquisition strategies, which has been underappreciated. Furthermore, the incomplete MEP pathways detected in some species suggest non-isoprenogenic roles and suggest that broader evolutionary pressures may shape the course of pathway utilization in closely related species, commensurate with their particular niche.

### Bacteria with dual isoprenoid biosynthesis strategies

In a starkly contrasting strategy, some bacteria possess complete copies of both the MEP and MEV pathways. Both pathways generate the same end products IDP and DMADP (Fig. 1), suggesting that dual pathway-encoding bacteria can utilize either for isoprenoid biosynthesis. However, each pathway may have specialized expression conditions, multifunctional intermediates, or roles in broader cellular processes.

The genus *Streptomyces*, and more broadly the Actinomycotota phylum, is well-known for producing a wide array of natural products, including antimicrobials, pharmaceuticals, and other economically relevant isoprenoids (88). Some *Streptomyces* possess genes for both the MEP and MEV pathways, which have each been demonstrated to be functional (89–92). *Streptomyces* genomes are rich in biosynthetic gene clusters (BGCs), genomic islands in which genes that catalyze the production of specialized metabolites are colocalized. Genes of the MEV pathway are sometimes found in such BGCs, and temporal expression of the MEV pathway has been observed to coincide with specialized natural product synthesis, suggesting that this pathway is being utilized in the production of the BGC end product (92, 93). Taken together, *Streptomyces* that possess both the MEP and MEV pathways appear to have distinct uses for each, despite generating identical end products. This functional partitioning allows the bacteria to tailor their metabolism during natural product synthesis (92).

Another case of dual pathway-encoding actinobacteria is found in the genus *Mycobacteria*, which contains the important human pathogen *M. tuberculosis*. While all *Mycobacteria* encode the MEP pathway, the *Mycobacterium ulcerans-Mycobacterium marinum* clade (MuMC) encodes both isoprenoid biosynthesis pathways (94, 95). In *M. marinum*, the MEP pathway has been determined to be essential, while the non-essential MEV pathway supports metabolic flexibility during stress (96). Interestingly, while the MEV pathway appears to be intact in *M. marinum*, the *M. ulcerans* pathway is likely nonfunctional due to a disruption of *hmgS* (95). The presence of a functional MEV pathway in *M. marinum* and loss of that functionality in the closely related *M. ulcerans* further underscores that each pathway is distinct and subject to selective pressure, although they both produce IDP. It is likely that the selective pressures are driven by the non-isoprenogenic differences of the MEP and MEV pathways.

In contrast to *Mycobacteria*, the genus *Listeria* primarily uses MEV with some species possessing an incomplete, likely non-isoprenogenic MEP pathway (11, 77, 97). As discussed in “The MEP and MEV pathways for isoprenoid biosynthesis,” above, *L. monocytogenes* and *L. innocua* have incomplete MEP pathways that terminate at the intermediate MEcDP, which has been implicated in oxidative stress signaling (12, 77). The termination of this partial pathway at MEcDP synthesis, in contrast to primary IDP synthesis via the MEV pathway, suggests that this could be the root of the evolutionary pressure for *Listeria* to retain an incomplete MEP pathway. Taken together, encoding both pathways partially or in full enables bacteria to fine-tune isoprenoid synthesis under specific conditions or even to generate intermediates that might protect them from environmental stress.

### Intra-genus diversity in isoprenoid acquisition strategies

When bacterial phylogeny is overlaid with isoprenoid pathway usage data, a striking pattern of heterogeneity emerges where phyla and isoprenoid acquisition strategy do not obviously align (Fig. 2A). We performed genus-level pathway analysis and confirmed that the MEP pathway is the predominant bacterial isoprenoid biosynthesis pathway, although there are lineages that use the MEV pathway. Interestingly, the phyla Actinomycetota and Bacillota contain considerable diversity compared to the other represented phyla (Fig. 2A). Isoprenoid biosynthesis gene presence-or-absence analysis was also performed for 4,249 bacterial species representing 1,412 genera (Fig. 2B; Table S1). We refer the reader to this supplementary resource for species-level information regarding which isoprenoid genes were identified. In summary, for the species analyzed, 82.94% use MEP, 12.03% use MEV, 2.42% have both pathways, and 2.61% have neither (Table S1).

Isoprenoid biosynthesis genes may be acquired in a lineage through a variety of mechanisms, including vertical inheritance from a common ancestor, horizontal transfer from another species, or by some combination of both. The distribution of pathways and punctate nodes of heterogeneity observed in Fig. 2A suggests multiple routes of diversification for these pathways. MEP genes are not found in operons and are extensively distributed across all phyla (Fig. 2A), making it unlikely that their distribution is a result of HGT (98). Conversely, MEV genes are more frequently found in operons, suggesting that some occasions of pathway diversity result from HGT.

Within individual genera, patterns emerge that suggest potential pathway decay. Of the 110 *Wolbachia* species analyzed, most encode MEP genes, while less than 2% have neither pathway (Table S1), potentially suggesting pathway loss from the genus ancestor. Around 8% of *Streptococcus* species and approximately half of *Enterococcus* species analyzed have an incomplete MEP pathway in addition to a complete MEV pathway (Table S1), potentially representing another example of a partial MEP pathway acting as an oxidative stress sensor as discussed in “The MEP and MEV pathways for isoprenoid biosynthesis,” above. Furthermore, while *Legionella* have been characterized as encoding the MEV pathway, all *Legionella* species analyzed lack the phosphomevalonate kinase PmvK, and most species additionally lack the hydroxymethylglutaryl-CoA synthase HmgS (99). Previous assignments may have relied on single gene presence-or-absence data; however, because these *Legionella* lack one or more critical enzymes in this pathway, they did not meet our threshold to be assigned as encoding the MEV pathway. Further work is needed to address whether alternative enzymes exist to compensate for the lack of canonical mevalonate enzymes or how *Legionella* otherwise acquires isoprenoid precursors in their absence. Taken together, these examples present fascinating questions as to whether these are cases of specific gene loss to accumulate a particular intermediate or whether these lineages are shedding an entire pathway.

Alternatively, in some clades, the pattern of distribution suggests pathway acquisition. While most members of the phylum Bacteriodota encode MEP, a considerable subset of related genera instead encodes only the MEV pathway, suggesting that a common ancestor of this group may have acquired MEV or potentially even retained an ancestral MEV pathway (Fig. 2A) (58). This gene acquisition is also observed in *Burkholderia* and *Clostridium*, which are largely MEP-encoding but have very rare instances of species with genes for both pathways (~4% of each genus have both, Table S1). When genera are analyzed for diversity in isoprenoid acquisition strategies, over half of the instances of intra-genus diversity were due to species encoding multiple genes for both pathways (25/44 genera, Table S1).

Finally, some genera have such varied pathway usage that it becomes difficult to speculate on an ancestral pathway route. In the case of the order Rickettsiales, there are indications of both vertical inheritance and horizontal gene transfer. Some genera within Rickettsiales possess a complete MEP pathway while others encode only downstream genes to process imported IDP, suggesting vertical inheritance and pathway degradation (86). Meanwhile, Rickettsiales that lack a complete MEP pathway often have Idi, which is hypothesized to have been acquired horizontally from non-alphaproteobacterial origins (87). Furthermore, nearly the full breadth of pathway diversity observed in the bacterial kingdom can be found in the genus *Brachybacterium*. The majority of the species analyzed encode genes for the MEP pathway, while the closely related *Bacillus halotolerans* and *Brachybacterium kimchii* encode genes only for the MEV pathway, and *Bordetella avium* encodes genes for both pathways (Fig. 2C). *Brachybacterium* species have been isolated from a wide range of niches, including associations with animals, soil, fresh and seawater, dairy, seafood, and, rarely, as pathogens (100). The wide variety of isoprenoid biosynthesis strategies detected in this genus may reflect the broad niche range of its species and suggest that yet-undetermined evolutionary pressures may be shaping pathway utilization across niches. Further work is needed to understand the evolutionary pressures that influence pathway suitability for a given niche and, in particular, why *Brachybacterium* species possess a range of isoprenoid biosynthesis strategies.

It is important to note that these analyses examine isoprenoid biosynthesis at the genome level, that is, these suggested routes of diversification are based on homologous gene presence-or-absence data and require experimental validation. For example, several *Streptomyces* species analyzed encode HmgR from the MEV pathway in addition to a complete MEP pathway, necessitating further study to determine whether a single gene from the other pathway contributes to isoprenoid biosynthesis (Table S1). Nonetheless, by taking a comprehensive view of isoprenoid biosynthesis through the lens of evolution, it becomes clear how evolution acts not only at a species level but also at the level of individual genes and metabolites. The MEP and MEV pathways both fulfill a requirement for cells to generate essential isoprenoid precursors, but each uses unique mechanisms and energetics, which may influence which pathway is selected for in a given niche. Although all bacteria must possess either or both of these pathways—or steal from a host—there is great flexibility for species to utilize the pathway best suited for their niche.

## SYNTHETIC BIOLOGY APPROACHES TO ENGINEER ISOPRENOID BIOSYNTHESIS

Economically relevant isoprenoids are critical to many industries, including agriculture, pharmaceuticals, and fuels, and are largely generated via unsustainable practices (101, 102). High demand and current unsustainable production methods have motivated synthetic biology efforts toward a “microbial cell factory” approach to replace or supplement current methods of isoprenoid synthesis (103–105). For example, the need for large-scale petroleum processing can be reduced or eliminated by engineering microbes to produce isoprene and other “platform chemicals,” which serve as building blocks for high-value products (102, 106, 107). Drugs like the antimalarial artemisinin and the anticancer paclitaxel, which were originally isolated from slow-growing plants, can be produced in microbes at much higher yields (108, 109). Additional efforts are underway to develop novel, non-natural isoprenoids with unique properties (110). Altogether, there have been significant and successful efforts to engineer microbes to produce platform chemicals, pharmaceuticals, fuels, and other valuable isoprenoids (106). A deeper understanding of microbial isoprenoid biosynthesis and its regulation will continue to be instrumental in the development of sustainable isoprenoid production technologies.

Through many years of research, the field has moved toward a clearer understanding of effective strategies to engineer microbes for isoprenoid production. Generally, engineering has focused on optimizing the expression of key enzymes for improved flux, ensuring sufficient precursor supply, and blocking competing pathways (111). Some of the highest isoprenoid yields of target molecules are from engineering the MEV pathway in microbes and yeast (104, 112–116). Generally, HmgR, Mvk, and Idi represent the critical enzymatic steps targeted in MEV engineering approaches, although efforts must be tailored to each host organism and target molecule (113, 114, 117, 118). In contrast, engineering approaches addressing the MEP pathway have generally produced lower product yields than those of the MEV pathway, despite the greater theoretical yield of the MEP pathway (54). A critical barrier to successful MEP pathway engineering is an incomplete understanding of how to circumvent the tight regulation of this pathway, which includes feedback inhibition at the key regulatory step mediated by Dxs (119–121). Another challenge in MEP pathway engineering lies in the enzymes IspG and IspH, which must be loaded with iron-sulfur clusters, supplied with reducing equivalents to function, and are notoriously oxygen sensitive (122).

One strategy to increase terpenoid yield is to express both isoprenoid biosynthetic pathways in one organism, although such heterologous expression is limited by imbalanced reducing equivalents, unidentified regulatory networks, and distinct cofactors and energetic requirements. Exogenous MEP expression in an organism that natively uses the MEV pathway is possible, although it remains limited by yield and requires the support of multiple partner enzymes (9, 123–125). For example, MEP expression in yeast has not produced a higher yield of target terpenoid products despite the MEP pathway having a higher theoretical productivity (126). Conversely, exogenous MEV expression in organisms natively using MEP has led to enhanced isoprenoid yields, with some groups demonstrating synergy between the two pathways (14, 104, 127). This improved yield upon expression of both pathways may be due to the generation of reducing power by the MEV pathway (Fig. 1; Table 1) or by the relief of feedback inhibition due to increased overall flux.

There is much to be learned from the dual-isoprenoid biosynthesis pathway-bearing organisms that can help metabolic engineers unlock greater product yields. For example, there is evidence for metabolic compensation in a MEV mutant of *M. marinum*, which encodes both pathways, although interestingly, MEV pathway overexpression did not compensate for a loss of MEP (96). Further study of such interactions is critical to reveal synergistic properties or non-isoprenogenic roles of the MEP or MEV pathways in these species, advancing the development of engineered microbes for sustainable isoprenoid production. By understanding the extensive heterogeneity generated through natural bacterial evolution, engineering approaches can skip the arduous process of developing and optimizing a novel biosynthetic route, instead utilizing the existing strategies in nature.

## CONCLUDING REMARKS

In this review, we argue that the extensive diversity observed at the levels of enzyme isoforms, pathway usage, and pathway completeness necessitates a more nuanced view of evolutionary inheritance of isoprenoid biosynthesis, as well as an appreciation of non-isoprenogenic effects of these pathways.

Each pathway has unique energetic features that may influence its acquisition or retention. The MEP pathway is more carbon-efficient, while the MEV pathway is more energy-efficient, suggesting that some bacteria encode different pathways to meet different energetic needs dictated by varied host niches and environmental challenges. While we cannot at this time define the exact evolutionary pressures influencing whether a bacterial species will encode one pathway, both, or neither, it seems likely that IDP synthesis strategies diversified among bacteria via a variety of mechanisms.

Finally, the heterogeneity observed across bacteria presents a fascinating perspective of the “natural engineering” achieved through evolution and offers insights to rationally design and optimize isoprenoid metabolism to tackle real-world sustainability issues. The extensive heterogeneity of pathway usage, enzyme forms, and end products that exist in nature represents an abundant source of natural diversity that can be harnessed to achieve such engineering goals.

In this review, we have examined the distribution of pathways across bacteria and highlighted previously underappreciated intra-genus diversity, likely owing to a variety of factors including environmental and host niches, as well as yet-undetermined evolutionary pressures. Analysis of natural instances of isoprenoid biosynthesis diversity, especially of those bacteria with incomplete or both pathways, provides an opportunity to further understand what roles the MEP and MEV pathways have beyond IDP generation. These roles may include oxidative stress signaling or other off-target effects of intermediate metabolites yet to be discovered. These non-isoprenogenic roles may be part of the evolutionary pressures that affect species’ acquisition or retention of one path over the other. The continued study of isoprenoid heterogeneity among bacteria will be instrumental in the development of microbes for sustainable isoprenoid production.

## SUPPLEMENTAL MATERIAL

Table S1 jb.00441-25-s0001.xlsx Pathway utilization by species (A) or genus (B).

ASM does not own the copyrights to Supplemental Material that may be linked to, or accessed through, an article. The authors have granted ASM a non-exclusive, world-wide license to publish the Supplemental Material files. Please contact the corresponding author directly for reuse.

## References

[1] Faylo JL, Ronnebaum TA, Christianson DW. 2021. Assembly-line catalysis in bifunctional terpene synthases. Acc Chem Res 54:3780–3791. doi:10.1021/acs.accounts.1c00296 34254507 PMC8530900

[2] Holstein SA, Hohl RJ. 2004. Isoprenoids: remarkable diversity of form and function. Lipids 39:293–309. doi:10.1007/s11745-004-1233-3 15357017

[3] Lange BM, Rujan T, Martin W, Croteau R. 2000. Isoprenoid biosynthesis: the evolution of two ancient and distinct pathways across genomes. Proc Natl Acad Sci USA 97:13172–13177. doi:10.1073/pnas.240454797 11078528 PMC27197

[4] Avalos M, Garbeva P, Vader L, van Wezel GP, Dickschat JS, Ulanova D. 2022. Biosynthesis, evolution and ecology of microbial terpenoids. Nat Prod Rep 39:249–272. doi:10.1039/D1NP00047K 34612321

[5] Goldstein JL, Brown MS. 1990. Regulation of the mevalonate pathway. Nature 343:425–430. doi:10.1038/343425a0 1967820

[6] Park J, Matralis AN, Berghuis AM, Tsantrizos YS. 2014. Human isoprenoid synthase enzymes as therapeutic targets. Front Chem 2:50. doi:10.3389/fchem.2014.00050 25101260 PMC4106277

[7] Lombard J, López-García P, Moreira D. 2012. Phylogenomic investigation of phospholipid synthesis in archaea. Archaea 2012:630910. doi:10.1155/2012/630910 23304072 PMC3533463

[8] González-Hernández RA, Valdez-Cruz NA, Macías-Rubalcava ML, Trujillo-Roldán MA. 2023. Overview of fungal terpene synthases and their regulation. World J Microbiol Biotechnol 39:194. doi:10.1007/s11274-023-03635-y 37169980 PMC10175467

[9] Kirby J, Keasling JD. 2009. Biosynthesis of plant isoprenoids: perspectives for microbial engineering. Annu Rev Plant Biol 60:335–355. doi:10.1146/annurev.arplant.043008.091955 19575586

[10] Pérez-Gil J, Rodríguez-Concepción M. 2013. Metabolic plasticity for isoprenoid biosynthesis in bacteria. Biochem J 452:19–25. doi:10.1042/BJ20121899 23614721

[11] Heuston S, Begley M, Gahan CGM, Hill C. 2012. Isoprenoid biosynthesis in bacterial pathogens. Microbiology (Reading) 158:1389–1401. doi:10.1099/mic.0.051599-0 22466083

[12] Perez-Gil J, Behrendorff J, Douw A, Vickers CE. 2024. The methylerythritol phosphate pathway as an oxidative stress sense and response system. Nat Commun 15:5303. doi:10.1038/s41467-024-49483-8 38906898 PMC11192765

[13] Chen ZW. 2013. Multifunctional immune responses of HMBPP-specific Vγ2Vδ2 T cells in M. tuberculosis and other infections. Cell Mol Immunol 10:58–64. doi:10.1038/cmi.2012.46 23147720 PMC3664056

[14] Yang C, Gao X, Jiang Y, Sun B, Gao F, Yang S. 2016. Synergy between methylerythritol phosphate pathway and mevalonate pathway for isoprene production in Escherichia coli. Metab Eng 37:79–91. doi:10.1016/j.ymben.2016.05.003 27174717

[15] Gruchattka E, Hädicke O, Klamt S, Schütz V, Kayser O. 2013. In silico profiling of Escherichia coli and Saccharomyces cerevisiae as terpenoid factories. Microb Cell Fact 12:84. doi:10.1186/1475-2859-12-84 24059635 PMC3852115

[16] Parks DH, Chuvochina M, Waite DW, Rinke C, Skarshewski A, Chaumeil P-A, Hugenholtz P. 2018. A standardized bacterial taxonomy based on genome phylogeny substantially revises the tree of life. Nat Biotechnol 36:996–1004. doi:10.1038/nbt.4229 30148503

[17] Letunic I, Bork P. 2021. Interactive Tree Of Life (iTOL) v5: an online tool for phylogenetic tree display and annotation. Nucleic Acids Res 49:W293–W296. doi:10.1093/nar/gkab301 33885785 PMC8265157

[18] Marshall B, Amritkar K, Wolfe M, Kaçar B, Landick R. 2023. Evolutionary flexibility and rigidity in the bacterial methylerythritol phosphate (MEP) pathway. Front Microbiol 14:1286626. doi:10.3389/fmicb.2023.1286626 38029103 PMC10663253

[19] Sayers EW, Bolton EE, Brister JR, Canese K, Chan J, Comeau DC, Connor R, Funk K, Kelly C, Kim S, Madej T, Marchler-Bauer A, Lanczycki C, Lathrop S, Lu Z, Thibaud-Nissen F, Murphy T, Phan L, Skripchenko Y, Tse T, Wang J, Williams R, Trawick BW, Pruitt KD, Sherry ST. 2022. Database resources of the national center for biotechnology information. Nucleic Acids Res 50:D20–D26. doi:10.1093/nar/gkab1112 34850941 PMC8728269

[20] Edgar RC. 2004. MUSCLE: multiple sequence alignment with high accuracy and high throughput. Nucleic Acids Res 32:1792–1797. doi:10.1093/nar/gkh340 15034147 PMC390337

[21] Guindon S, Dufayard J-F, Lefort V, Anisimova M, Hordijk W, Gascuel O. 2010. New algorithms and methods to estimate maximum-likelihood phylogenies: assessing the performance of PhyML 3.0. Syst Biol 59:307–321. doi:10.1093/sysbio/syq010 20525638

[22] Lois LM, Campos N, Putra SR, Danielsen K, Rohmer M, Boronat A. 1998. Cloning and characterization of a gene from Escherichia coli encoding a transketolase-like enzyme that catalyzes the synthesis of d-1-deoxyxylulose 5-phosphate, a common precursor for isoprenoid, thiamin, and pyridoxol biosynthesis. Proc Natl Acad Sci USA 95:2105–2110. doi:10.1073/pnas.95.5.2105 9482846 PMC19265

[23] Banerjee A, Wu Y, Banerjee R, Li Y, Yan H, Sharkey TD. 2013. Feedback inhibition of deoxy-d-xylulose-5-phosphate synthase regulates the methylerythritol 4-phosphate pathway*. J Biol Chem 288:16926–16936. doi:10.1074/jbc.M113.464636 23612965 PMC3675625

[24] Patel H, Nemeria NS, Brammer LA, Freel Meyers CL, Jordan F. 2012. Observation of thiamin-bound intermediates and microscopic rate constants for their interconversion on 1‑deoxy‑d‑xylulose 5‑phosphate synthase: 600-fold rate acceleration of pyruvate decarboxylation by d‑glyceraldehyde-3-phosphate. J Am Chem Soc 134:18374–18379. doi:10.1021/ja307315u 23072514 PMC3494461

[25] Sangari FJ, Pérez-Gil J, Carretero-Paulet L, García-Lobo JM, Rodríguez-Concepción M. 2010. A new family of enzymes catalyzing the first committed step of the methylerythritol 4-phosphate (MEP) pathway for isoprenoid biosynthesis in bacteria. Proc Natl Acad Sci USA 107:14081–14086. doi:10.1073/pnas.1001962107 20660776 PMC2922546

[26] Kuzuyama T, Shimizu T, Takahashi S, Seto H. 1998. Fosmidomycin, a specific inhibitor of 1-deoxy-d-xylulose 5-phosphate reductoisomerase in the nonmevalonate pathway for terpenoid biosynthesis. Tetrahedron Lett 39:7913–7916. doi:10.1016/S0040-4039(98)01755-9

[27] Rodríguez-Concepción M, Boronat A. 2002. Elucidation of the methylerythritol phosphate pathway for isoprenoid biosynthesis in bacteria and plastids. A metabolic milestone achieved through genomics. Plant Physiol 130:1079–1089. doi:10.1104/pp.007138 12427975 PMC1540259

[28] Testa CA, Lherbet C, Pojer F, Noel JP, Poulter CD. 2006. Cloning and expression of IspDF from Mesorhizobium loti. Characterization of a bifunctional protein that catalyzes non-consecutive steps in the methylerythritol phosphate pathway. Biochim Biophys Acta 1764:85–96. doi:10.1016/j.bbapap.2005.08.006 16203191

[29] Bitok JK, Meyers CF. 2012. 2C-Methyl-d-erythritol 4-phosphate enhances and sustains cyclodiphosphate synthase IspF activity. ACS Chem Biol 7:1702–1710. doi:10.1021/cb300243w 22839733 PMC3477264

[30] Eberl M, Hintz M, Reichenberg A, Kollas A-K, Wiesner J, Jomaa H. 2003. Microbial isoprenoid biosynthesis and human γδ T cell activation. FEBS Lett 544:4–10. doi:10.1016/s0014-5793(03)00483-6 12782281

[31] Artsatbanov Vy, Vostroknutova GN, Shleeva MO, Goncharenko AV, Zinin AI, Ostrovsky DN, Kapreliants AS. 2012. Influence of oxidative and nitrosative stress on accumulation of diphosphate intermediates of the non-mevalonate pathway of isoprenoid biosynthesis in corynebacteria and mycobacteria. Biochemistry (Mosc) 77:362–371. doi:10.1134/S0006297912040074 22809155

[32] Banerjee A, Sharkey TD. 2014. Methylerythritol 4-phosphate (MEP) pathway metabolic regulation. Nat Prod Rep 31:1043–1055. doi:10.1039/c3np70124g 24921065

[33] Rohdich F, Hecht S, Gärtner K, Adam P, Krieger C, Amslinger S, Arigoni D, Bacher A, Eisenreich W. 2002. Studies on the nonmevalonate terpene biosynthetic pathway: metabolic role of IspH (LytB) protein. Proc Natl Acad Sci USA 99:1158–1163. doi:10.1073/pnas.032658999 11818558 PMC122160

[34] Bongers M, Perez-Gil J, Hodson MP, Schrübbers L, Wulff T, Sommer MO, Nielsen LK, Vickers CE. 2020. Adaptation of hydroxymethylbutenyl diphosphate reductase enables volatile isoprenoid production. eLife 9:e48685. doi:10.7554/eLife.48685 32163032 PMC7067565

[35] Huang S, Xue Y, Ma Y, Zhou C. 2022. Microbial (E)-4-hydroxy-3-methylbut-2-enyl pyrophosphate reductase (IspH) and its biotechnological potential: a mini review. Front Bioeng Biotechnol 10:1057938. doi:10.3389/fbioe.2022.1057938 36524053 PMC9745026

[36] Berthelot K, Estevez Y, Deffieux A, Peruch F. 2012. Isopentenyl diphosphate isomerase: a checkpoint to isoprenoid biosynthesis. Biochimie 94:1621–1634. doi:10.1016/j.biochi.2012.03.021 22503704

[37] Wang C-H, Hou J, Deng H-K, Wang L-J. 2023. Microbial production of mevalonate. J Biotechnol 370:1–11. doi:10.1016/j.jbiotec.2023.05.005 37209831

[38] Kutschera U, Niklas KJ. 2005. Endosymbiosis, cell evolution, and speciation. Theory Biosci 124:1–24. doi:10.1016/j.thbio.2005.04.001 17046345

[39] Miziorko HM. 2011. Enzymes of the mevalonate pathway of isoprenoid biosynthesis. Arch Biochem Biophys 505:131–143. doi:10.1016/j.abb.2010.09.028 20932952 PMC3026612

[40] Hoshino Y, Villanueva L. 2023. Four billion years of microbial terpenome evolution. FEMS Microbiol Rev 47:fuad008. doi:10.1093/femsre/fuad008 36941124

[41] Hedl M, Tabernero L, Stauffacher CV, Rodwell VW. 2004. Class II 3-hydroxy-3-methylglutaryl coenzyme A reductases. J Bacteriol 186:1927–1932. doi:10.1128/JB.186.7.1927-1932.2004 15028676 PMC374403

[42] Miller BR, Kung Y. 2018. Structural features and domain movements controlling substrate binding and cofactor specificity in class II HMG-CoA reductase. Biochemistry 57:654–662. doi:10.1021/acs.biochem.7b00999 29224355 PMC6170158

[43] Hinson DD, Chambliss KL, Toth MJ, Tanaka RD, Gibson KM. 1997. Post-translational regulation of mevalonate kinase by intermediates of the cholesterol and nonsterol isoprene biosynthetic pathways. J Lipid Res 38:2216–2223. 9392419

[44] Voynova NE, Rios SE, Miziorko HM. 2004. Staphylococcus aureus mevalonate kinase: isolation and characterization of an enzyme of the isoprenoid biosynthetic pathway. J Bacteriol 186:61–67. doi:10.1128/JB.186.1.61-67.2004 14679225 PMC303434

[45] Andreassi JL, Dabovic K, Leyh TS. 2004. Streptococcus pneumoniae isoprenoid biosynthesis is downregulated by diphosphomevalonate: an antimicrobial target. Biochemistry 43:16461–16466. doi:10.1021/bi048075t 15610040

[46] Kuzuyama T, Seto H. 2012. Two distinct pathways for essential metabolic precursors for isoprenoid biosynthesis. Proc Jpn Acad, Ser B 88:41–52. doi:10.2183/pjab.88.41 22450534 PMC3365244

[47] McFadden GI. 2001. Primary and secondary endosymbiosis and the origin of plastids. J Phycol 37:951–959. doi:10.1046/j.1529-8817.2001.01126.x

[48] Margulis L. 1970. Origin of eukaryotic cells. Yale University Press, New Haven.

[49] van Dooren GG, Striepen B. 2013. The algal past and parasite present of the apicoplast. Annu Rev Microbiol 67:271–289. doi:10.1146/annurev-micro-092412-155741 23808340

[50] Zhao L, Chang W, Xiao Y, Liu H, Liu P. 2013. Methylerythritol phosphate pathway of isoprenoid biosynthesis. Annu Rev Biochem 82:497–530. doi:10.1146/annurev-biochem-052010-100934 23746261 PMC5031371

[51] Gräwert T, Groll M, Rohdich F, Bacher A, Eisenreich W. 2011. Biochemistry of the non-mevalonate isoprenoid pathway. Cell Mol Life Sci 68:3797–3814. doi:10.1007/s00018-011-0753-z 21744068 PMC11114746

[52] Hunter WN. 2011. Isoprenoid precursor biosynthesis offers potential targets for drug discovery against diseases caused by apicomplexan parasites. Curr Top Med Chem 11:2048–2059. doi:10.2174/156802611796575867 21619509 PMC3466564

[53] Vranová E, Coman D, Gruissem W. 2013. Network analysis of the MVA and MEP pathways for isoprenoid synthesis. Annu Rev Plant Biol 64:665–700. doi:10.1146/annurev-arplant-050312-120116 23451776

[54] Volke DC, Rohwer J, Fischer R, Jennewein S. 2019. Investigation of the methylerythritol 4-phosphate pathway for microbial terpenoid production through metabolic control analysis. Microb Cell Fact 18:192. doi:10.1186/s12934-019-1235-5 31690314 PMC6833178

[55] Di X, Ortega-Alarcon D, Kakumanu R, Iglesias-Fernandez J, Diaz L, Baidoo EEK, Velazquez-Campoy A, Rodríguez-Concepción M, Perez-Gil J. 2023. MEP pathway products allosterically promote monomerization of deoxy-D-xylulose-5-phosphate synthase to feedback-regulate their supply. Plant Commun 4:100512. doi:10.1016/j.xplc.2022.100512 36575800 PMC10203381

[56] Brown AC, Eberl M, Crick DC, Jomaa H, Parish T. 2010. The nonmevalonate pathway of isoprenoid biosynthesis in Mycobacterium tuberculosis is essential and transcriptionally regulated by Dxs. J Bacteriol 192:2424–2433. doi:10.1128/JB.01402-09 20172995 PMC2863480

[57] Rivasseau C, Seemann M, Boisson A-M, Streb P, Gout E, Douce R, Rohmer M, Bligny R. 2009. Accumulation of 2-C-methyl-d-erythritol 2,4-cyclodiphosphate in illuminated plant leaves at supraoptimal temperatures reveals a bottleneck of the prokaryotic methylerythritol 4-phosphate pathway of isoprenoid biosynthesis. Plant Cell Environ 32:82–92. doi:10.1111/j.1365-3040.2008.01903.x 19021881

[58] Lombard J, Moreira D. 2011. Origins and early evolution of the mevalonate pathway of isoprenoid biosynthesis in the three domains of life. Mol Biol Evol 28:87–99. doi:10.1093/molbev/msq177 20651049

[59] Matsumi R, Atomi H, Driessen AJM, van der Oost J. 2011. Isoprenoid biosynthesis in Archaea – biochemical and evolutionary implications. Res Microbiol 162:39–52. doi:10.1016/j.resmic.2010.10.003 21034816

[60] Boucher Y, Kamekura M, Doolittle WF. 2004. Origins and evolution of isoprenoid lipid biosynthesis in archaea. Mol Microbiol 52:515–527. doi:10.1111/j.1365-2958.2004.03992.x 15066037

[61] Guerra B, Recio C, Aranda-Tavío H, Guerra-Rodríguez M, García-Castellano JM, Fernández-Pérez L. 2021. The mevalonate pathway, a metabolic target in cancer therapy. Front Oncol 11:626971. doi:10.3389/fonc.2021.626971 33718197 PMC7947625

[62] Donald KA, Hampton RY, Fritz IB. 1997. Effects of overproduction of the catalytic domain of 3-hydroxy-3-methylglutaryl coenzyme A reductase on squalene synthesis in Saccharomyces cerevisiae. Appl Environ Microbiol 63:3341–3344. doi:10.1128/aem.63.9.3341-3344.1997 9292983 PMC168639

[63] Zhu P, Hou J, Xiong Y, Xie R, Wang Y, Wang F. 2024. Expanded archaeal genomes shed new light on the evolution of isoprenoid biosynthesis. Microorganisms 12:707. doi:10.3390/microorganisms12040707 38674651 PMC11052028

[64] Kanno K, Kuriki R, Yasuno Y, Shinada T, Ito T, Hemmi H. 2024. Archaeal mevalonate pathway in the uncultured bacterium Candidatus Promineifilum breve belonging to the phylum Chloroflexota. Appl Environ Microbiol 90:e0110624. doi:10.1128/aem.01106-24 39082809 PMC11337835

[65] Dellas N, Thomas ST, Manning G, Noel JP. 2013. Discovery of a metabolic alternative to the classical mevalonate pathway. eLife 2:e00672. doi:10.7554/eLife.00672 24327557 PMC3857490

[66] Carretero-Paulet L, Lipska A, Pérez-Gil J, Sangari FJ, Albert VA, Rodríguez-Concepción M. 2013. Evolutionary diversification and characterization of the eubacterial gene family encoding DXR type II, an alternative isoprenoid biosynthetic enzyme. BMC Evol Biol 13:180. doi:10.1186/1471-2148-13-180 24004839 PMC3847144

[67] Hoshino Y, Gaucher EA. 2018. On the origin of isoprenoid biosynthesis. Mol Biol Evol 35:2185–2197. doi:10.1093/molbev/msy120 29905874 PMC6107057

[68] Primak YA, Du M, Miller MC, Wells DH, Nielsen AT, Weyler W, Beck ZQ. 2011. Characterization of a feedback-resistant mevalonate kinase from the archaeon Methanosarcina mazei. Appl Environ Microbiol 77:7772–7778. doi:10.1128/AEM.05761-11 21908638 PMC3209144

[69] Kazieva E, Yamamoto Y, Tajima Y, Yokoyama K, Katashkina J, Nishio Y. 2017. Characterization of feedback-resistant mevalonate kinases from the methanogenic archaeons Methanosaeta concilii and Methanocella paludicola. Microbiology (Reading) 163:1283–1291. doi:10.1099/mic.0.000510 28869407 PMC5817203

[70] Hayakawa H, Motoyama K, Sobue F, Ito T, Kawaide H, Yoshimura T, Hemmi H. 2018. Modified mevalonate pathway of the archaeon Aeropyrum pernix proceeds via trans-anhydromevalonate 5-phosphate. Proc Natl Acad Sci USA 115:10034–10039. doi:10.1073/pnas.1809154115 30224495 PMC6176645

[71] Sauret-Güeto S, Urós EM, Ibáñez E, Boronat A, Rodríguez-Concepción M. 2006. A mutant pyruvate dehydrogenase E1 subunit allows survival of Escherichia coli strains defective in 1-deoxy-d-xylulose 5-phosphate synthase. FEBS Lett 580:736–740. doi:10.1016/j.febslet.2005.12.092 16414046

[72] Perez-Gil J, Uros EM, Sauret-Güeto S, Lois LM, Kirby J, Nishimoto M, Baidoo EEK, Keasling JD, Boronat A, Rodriguez-Concepcion M. 2012. Mutations in Escherichia coli aceE and ribB genes allow survival of strains defective in the first step of the isoprenoid biosynthesis pathway. PLoS One 7:e43775. doi:10.1371/journal.pone.0043775 22928031 PMC3424233

[73] Kirby J, Nishimoto M, Chow RWN, Baidoo EEK, Wang G, Martin J, Schackwitz W, Chan R, Fortman JL, Keasling JD. 2015. Enhancing terpene yield from sugars via novel routes to 1-deoxy-d-xylulose 5-phosphate. Appl Environ Microbiol 81:130–138. doi:10.1128/AEM.02920-14 25326299 PMC4272731

[74] Ostrovsky D, Shipanova I, Sibeldina L, Shashkov A, Kharatian E, Malyarova I, Tantsyrev G. 1992. A new cyclopyrophosphate as a bacterial antistressor? FEBS Lett 298:159–161. doi:10.1016/0014-5793(92)80045-i 1312021

[75] Ostrovsky D, Diomina G, Lysak E, Matveeva E, Ogrel O, Trutko S. 1998. Effect of oxidative stress on the biosynthesis of 2-C-methyl-d-erythritol-2,4-cyclopyrophosphate and isoprenoids by several bacterial strains. Arch Microbiol 171:69–72. doi:10.1007/s002030050680 9871022

[76] Begley M, Bron PA, Heuston S, Casey PG, Englert N, Wiesner J, Jomaa H, Gahan CGM, Hill C. 2008. Analysis of the isoprenoid biosynthesis pathways in Listeria monocytogenes reveals a role for the alternative 2-C-methyl-d-erythritol 4-phosphate pathway in murine infection. Infect Immun 76:5392–5401. doi:10.1128/IAI.01376-07 18765739 PMC2573353

[77] Lee ED, Navas KI, Portnoy DA. 2020. The nonmevalonate pathway of isoprenoid biosynthesis supports anaerobic growth of Listeria monocytogenes. Infect Immun 88:e00788-19. doi:10.1128/IAI.00788-19 31792073 PMC6977116

[78] Begley M, Gahan CGM, Kollas A-K, Hintz M, Hill C, Jomaa H, Eberl M. 2004. The interplay between classical and alternative isoprenoid biosynthesis controls γδ T cell bioactivity of Listeria monocytogenes. FEBS Lett 561:99–104. doi:10.1016/S0014-5793(04)00131-0 15013758

[79] Mains DR, Eallonardo SJ, Freitag NE. 2021. Identification of Listeria monocytogenes genes contributing to oxidative stress resistance under conditions relevant to host infection. Infect Immun 89:e00700-20. doi:10.1128/IAI.00700-20 33495274 PMC8090957

[80] Heuston S, Begley M, Davey MS, Eberl M, Casey PG, Hill C, Gahan CGM. 2012. HmgR, a key enzyme in the mevalonate pathway for isoprenoid biosynthesis, is essential for growth of Listeria monocytogenes EGDe. Microbiology (Reading, Engl) 158:1684–1693. doi:10.1099/mic.0.056069-0 22504435

[81] Gay L, Mezouar S, Cano C, Frohna P, Madakamutil L, Mège J-L, Olive D. 2022. Role of Vγ9vδ2 T lymphocytes in infectious diseases. Front Immunol 13:928441. doi:10.3389/fimmu.2022.928441 35924233 PMC9340263

[82] Sirand-Pugnet P, Citti C, Barré A, Blanchard A. 2007. Evolution of mollicutes: down a bumpy road with twists and turns. Res Microbiol 158:754–766. doi:10.1016/j.resmic.2007.09.007 18023150

[83] Boucher Y, Doolittle WF. 2000. The role of lateral gene transfer in the evolution of isoprenoid biosynthesis pathways. Mol Microbiol 37:703–716. doi:10.1046/j.1365-2958.2000.02004.x 10972794

[84] Yavari CA, Ramírez AS, Nicholas RAJ, Radford AD, Darby AC, Bradbury JM. 2017. Mycoplasma tullyi sp. nov., isolated from penguins of the genus Spheniscus. Int J Syst Evol Microbiol 67:3692–3698. doi:10.1099/ijsem.0.002052 28895509

[85] Tulman ER, Liao X, Szczepanek SM, Ley DH, Kutish GF, Geary SJ. 2012. Extensive variation in surface lipoprotein gene content and genomic changes associated with virulence during evolution of a novel North American house finch epizootic strain of Mycoplasma gallisepticum. Microbiology (Reading) 158:2073–2088. doi:10.1099/mic.0.058560-0 22628486

[86] Driscoll TP, Verhoeve VI, Guillotte ML, Lehman SS, Rennoll SA, Beier-Sexton M, Rahman MS, Azad AF, Gillespie JJ. 2017. Wholly Rickettsia! Reconstructed metabolic profile of the quintessential bacterial parasite of eukaryotic cells. mBio 8:e00859-17. doi:10.1128/mBio.00859-17 28951473 PMC5615194

[87] Ahyong V, Berdan CA, Burke TP, Nomura DK, Welch MD. 2019. A metabolic dependency for host isoprenoids in the obligate intracellular pathogen Rickettsia parkeri underlies a sensitivity to the statin class of host-targeted therapeutics. mSphere 4:e00536-19. doi:10.1128/mSphere.00536-19 31722991 PMC6854040

[88] Kuzuyama T. 2017. Biosynthetic studies on terpenoids produced by Streptomyces. J Antibiot 70:811–818. doi:10.1038/ja.2017.12 PMC5509993 28196976

[89] Takagi M, Kuzuyama T, Takahashi S, Seto H. 2000. A gene cluster for the mevalonate pathway from Streptomyces sp. strain CL190. J Bacteriol 182:4153–4157. doi:10.1128/JB.182.15.4153-4157.2000 10894721 PMC101890

[90] Kuzuyama T, Takagi M, Takahashi S, Seto H. 2000. Cloning and characterization of 1-deoxy-d-xylulose 5-phosphate synthase from Streptomyces sp. Strain CL190, which uses both the mevalonate and nonmevalonate pathways for isopentenyl diphosphate biosynthesis. J Bacteriol 182:891–897. doi:10.1128/JB.182.4.891-897.2000 10648511 PMC94361

[91] Seto H, Watanabe H, Furihata K. 1996. Simultaneous operation of the mevalonate and non-mevalonate pathways in the biosynthesis of isopentenly diphosphate in Streptomyces aeriouvifer. Tetrahedron Lett 37:7979–7982. doi:10.1016/0040-4039(96)01787-X

[92] Hamano Y, Dairi T, Yamamoto M, Kuzuyama T, Itoh N, Seto H. 2002. Growth-phase dependent expression of the mevalonate pathway in a terpenoid antibiotic-producing Streptomyces strain. Biosci Biotechnol Biochem 66:808–819. doi:10.1271/bbb.66.808 12036054

[93] Dairi T. 2005. Studies on biosynthetic genes and enzymes of isoprenoids produced by actinomycetes. J Antibiot 58:227–243. doi:10.1038/ja.2005.27 15981409

[94] Doig KD, Holt KE, Fyfe JAM, Lavender CJ, Eddyani M, Portaels F, Yeboah-Manu D, Pluschke G, Seemann T, Stinear TP. 2012. On the origin of Mycobacterium ulcerans, the causative agent of Buruli ulcer. BMC Genomics 13:258. doi:10.1186/1471-2164-13-258 22712622 PMC3434033

[95] Stinear TP, Seemann T, Pidot S, Frigui W, Reysset G, Garnier T, Meurice G, Simon D, Bouchier C, Ma L, Tichit M, Porter JL, Ryan J, Johnson PDR, Davies JK, Jenkin GA, Small PLC, Jones LM, Tekaia F, Laval F, Daffé M, Parkhill J, Cole ST. 2007. Reductive evolution and niche adaptation inferred from the genome of Mycobacterium ulcerans, the causative agent of Buruli ulcer. Genome Res 17:192–200. doi:10.1101/gr.5942807 17210928 PMC1781351

[96] Qabar CM, Baidoo EEK, Akyuz Turumtay E, Qayum TM, Keasling JD, Madigan CA, Portnoy DA, Cox JS. 2025. The mevalonate pathway of isoprenoid biosynthesis supports metabolic flexibility in Mycobacterium marinum. J Bacteriol 207:e0028725. doi:10.1128/jb.00287-25 41165411 PMC12632256

[97] Begley M, Gahan CGM, Kollas A-K, Hintz M, Hill C, Jomaa H, Eberl M. 2004. The interplay between classical and alternative isoprenoid biosynthesis controls gammadelta T cell bioactivity of Listeria monocytogenes. FEBS Lett 561:99–104. doi:10.1016/S0014-5793(04)00131-0 15013758

[98] Boucher Y, Douady CJ, Papke RT, Walsh DA, Boudreau MER, Nesbø CL, Case RJ, Doolittle WF. 2003. Lateral gene transfer and the origins of prokaryotic groups. Annu Rev Genet 37:283–328. doi:10.1146/annurev.genet.37.050503.084247 14616063

[99] Kanehisa M, Furumichi M, Sato Y, Matsuura Y, Ishiguro-Watanabe M. 2025. KEGG: biological systems database as a model of the real world. Nucleic Acids Res 53:D672–D677. doi:10.1093/nar/gkae909 39417505 PMC11701520

[100] de Castro I, Ribeiro S, Oliveira V, Coelho FJRC, de Lurdes Dapkevicius M, de Azevedo EB, Barcelos E Ramos J. 2023. Brachybacterium atlanticum sp. nov., a novel marine bacterium isolated from the Atlantic Ocean. Int J Syst Evol Microbiol 73. doi:10.1099/ijsem.0.005959 37540229

[101] Mosquera M, Jiménez G, Tabernero V, Vinueza-Vaca J, García-Estrada C, Kosalková K, Sola-Landa A, Monje B, Acosta C, Alonso R, Valera MÁ. 2021. Terpenes and terpenoids: building blocks to produce biopolymers. Sustain Chem 2:467–492. doi:10.3390/suschem2030026

[102] Leavell MD, McPhee DJ, Paddon CJ. 2016. Developing fermentative terpenoid production for commercial usage. Curr Opin Biotechnol 37:114–119. doi:10.1016/j.copbio.2015.10.007 26723008

[103] Moser S, Pichler H. 2019. Identifying and engineering the ideal microbial terpenoid production host. Appl Microbiol Biotechnol 103:5501–5516. doi:10.1007/s00253-019-09892-y 31129740 PMC6597603

[104] Kant G, Pandey A, Hasan A, Bux F, Kumari S, Srivastava S. 2024. Cell factories for methylerythritol phosphate pathway mediated terpenoid biosynthesis: an application of modern engineering towards sustainability. Process Biochem 139:146–164. doi:10.1016/j.procbio.2024.01.027

[105] Fordjour E, Mensah EO, Hao Y, Yang Y, Liu X, Li Y, Liu C-L, Bai Z. 2022. Toward improved terpenoids biosynthesis: strategies to enhance the capabilities of cell factories. Bioresour Bioprocess 9:6. doi:10.1186/s40643-022-00493-8 38647812 PMC10992668

[106] Hill P, Benjamin K, Bhattacharjee B, Garcia F, Leng J, Liu C-L, Murarka A, Pitera D, Rodriguez Porcel EM, da Silva I, Kraft C. 2020. Clean manufacturing powered by biology: how Amyris has deployed technology and aims to do it better. J Ind Microbiol Biotechnol 47:965–975. doi:10.1007/s10295-020-02314-3 33029730 PMC7695652

[107] Kant G, Pandey A, Shekhar H, Srivastava S. 2023. Enhanced bio-synthesis of isoprene via modifying mevalonate and methylerythritol phosphate pathways for industrial application: a review. Process Biochem 130:256–271. doi:10.1016/j.procbio.2023.04.021

[108] Benjamin KR, Silva IR, Cherubim JP, McPhee D, Paddon CJ. 2016. Developing commercial production of semi-synthetic artemisinin, and of β-farnesene, an isoprenoid produced by fermentation of Brazilian sugar. J Braz Chem Soc. doi:10.5935/0103-5053.20160119

[109] McElroy C, Jennewein S. 2018. Biotechnology of natural products, p 145–185

[110] Daletos G, Stephanopoulos G. 2020. Protein engineering strategies for microbial production of isoprenoids. Metab Eng Commun 11:e00129. doi:10.1016/j.mec.2020.e00129 32612930 PMC7322351

[111] Zhao S, Jia L, Tang H. 2025. Strategies for enhancing the yield of mevalonate in microbial strains. Theor Nat Sci 139:153–164. doi:10.54254/2753-8818/2025.AU27357

[112] Liu Y, Wang Z, Cui Z, Qi Q, Hou J. 2022. Progress and perspectives for microbial production of farnesene. Bioresour Technol 347:126682. doi:10.1016/j.biortech.2022.126682 35007732

[113] Navale GR, Dharne MS, Shinde SS. 2021. Metabolic engineering and synthetic biology for isoprenoid production in Escherichia coli and Saccharomyces cerevisiae. Appl Microbiol Biotechnol 105:457–475. doi:10.1007/s00253-020-11040-w 33394155

[114] Liu C-L, Xue K, Yang Y, Liu X, Li Y, Lee TS, Bai Z, Tan T. 2022. Metabolic engineering strategies for sesquiterpene production in microorganism. Crit Rev Biotechnol 42:73–92. doi:10.1080/07388551.2021.1924112 34256675

[115] Tang R, Wen Q, Li M, Zhang W, Wang Z, Yang J. 2021. Recent advances in the biosynthesis of farnesene using metabolic engineering. J Agric Food Chem 69:15468–15483. doi:10.1021/acs.jafc.1c06022 34905684

[116] Morrone D, Lowry L, Determan MK, Hershey DM, Xu M, Peters RJ. 2010. Increasing diterpene yield with a modular metabolic engineering system in E. coli: comparison of MEV and MEP isoprenoid precursor pathway engineering. Appl Microbiol Biotechnol 85:1893–1906. doi:10.1007/s00253-009-2219-x 19777230 PMC2811251

[117] Wang Q, Quan S, Xiao H. 2019. Towards efficient terpenoid biosynthesis: manipulating IPP and DMAPP supply. Bioresour Bioprocess 6:6. doi:10.1186/s40643-019-0242-z

[118] Li M, Hou F, Wu T, Jiang X, Li F, Liu H, Xian M, Zhang H. 2020. Recent advances of metabolic engineering strategies in natural isoprenoid production using cell factories. Nat Prod Rep 37:80–99. doi:10.1039/c9np00016j 31073570

[119] Khana DB, Tatli M, Rivera Vazquez J, Weraduwage SM, Stern N, Hebert AS, Angelica Trujillo E, Stevenson DM, Coon JJ, Sharky TD, Amador-Noguez D. 2023. Systematic analysis of metabolic bottlenecks in the methylerythritol 4-phosphate (MEP) pathway of Zymomonas mobilis. mSystems 8:e0009223. doi:10.1128/msystems.00092-23 36995223 PMC10134818

[120] Ma Y, McClure DD, Somerville MV, Proschogo NW, Dehghani F, Kavanagh JM, Coleman NV. 2019. Metabolic engineering of the MEP pathway in Bacillus subtilis for increased biosynthesis of menaquinone-7. ACS Synth Biol 8:1620–1630. doi:10.1021/acssynbio.9b00077 31250633

[121] Gao X, Gao F, Liu D, Zhang H, Nie X, Yang C. 2016. Engineering the methylerythritol phosphate pathway in cyanobacteria for photosynthetic isoprene production from CO_2_. Energy Environ Sci 9:1400–1411. doi:10.1039/C5EE03102H

[122] Zhou J, Yang L, Wang C, Choi E-S, Kim S-W. 2017. Enhanced performance of the methylerythritol phosphate pathway by manipulation of redox reactions relevant to IspC, IspG, and IspH. J Biotechnol 248:1–8. doi:10.1016/j.jbiotec.2017.03.005 28279816

[123] Partow S, Siewers V, Daviet L, Schalk M, Nielsen J. 2012. Reconstruction and evaluation of the synthetic bacterial MEP pathway in Saccharomyces cerevisiae. PLoS One 7:e52498. doi:10.1371/journal.pone.0052498 23285068 PMC3532213

[124] Carlsen S, Ajikumar PK, Formenti LR, Zhou K, Phon TH, Nielsen ML, Lantz AE, Kielland-Brandt MC, Stephanopoulos G. 2013. Heterologous expression and characterization of bacterial 2-C-methyl-d-erythritol-4-phosphate pathway in Saccharomyces cerevisiae. Appl Microbiol Biotechnol 97:5753–5769. doi:10.1007/s00253-013-4877-y 23636690

[125] Kirby J, Dietzel KL, Wichmann G, Chan R, Antipov E, Moss N, Baidoo EEK, Jackson P, Gaucher SP, Gottlieb S, LaBarge J, Mahatdejkul T, Hawkins KM, Muley S, Newman JD, Liu P, Keasling JD, Zhao L. 2016. Engineering a functional 1-deoxy-D-xylulose 5-phosphate (DXP) pathway in Saccharomyces cerevisiae. Metab Eng 38:494–503. doi:10.1016/j.ymben.2016.10.017 27989805 PMC5718835

[126] Whited GM, Feher FJ, Benko DA, Cervin MA, Chotani GK, McAuliffe JC, LaDuca RJ, Ben-Shoshan EA, Sanford KJ. 2010. Technology update: Development of a gas-phase bioprocess for isoprene-monomer production using metabolic pathway engineering. Ind Biotechnol (New Rochelle N Y) 6:152–163. doi:10.1089/ind.2010.6.152

[127] Martin VJJ, Pitera DJ, Withers ST, Newman JD, Keasling JD. 2003. Engineering a mevalonate pathway in Escherichia coli for production of terpenoids. Nat Biotechnol 21:796–802. doi:10.1038/nbt833 12778056

